# Double, Recurrent and Bilateral Tubal Pregnancies

**Published:** 1918-01

**Authors:** Aime Paul Heineck

**Affiliations:** Chicago


### Journal-Record of Medicine

Successor to Atlanta Medical and Surgical Journal, Established 1855
and Southern Medical Record, Established 1870

**OWNED BY THE ATLANTA MEDICAL JOURNAL COMPANY**

PUBLISHED MONTHLY

*Official Organ Fulton County Medical Society, State Examing Board, Atlanta, Birmingham and Atlantic Railroad Surgeon's Association, Chattahoochee Valley Medical and Surgical Association, Etc.*

A. W. STIRLING, M. D., C. M., D. P. H.; J. S. HURT, B. Ph., M.D.
GEO. M. NILES, M. D., W. J. LOVE, M. D., (Ala.); Associate Editors

E. W. ALLEN, Business Manager

**COLLABORATORS**

W. F. WESTMORELAND, M. D., General Surgery
F. W. McRAE, M. D., Abdominal Surgery
ALLEN H. BUNCE, Medical Societies
E. B. BLOCK, M. D., Diseases of the Nervous System
MICHAEL HOKE, M. D., Orthopedic Surgery
CYRUS W. STRICKLER, M. D., Legal Medicine and Medical Legislation
E. C. DAVIS, A. B., M. D., Obstetrics
E. G. JONES, A. B., M. D., Gynecology
ALLEN H. BUNCE, M. D., Pathology and Bacteriology
R. T. DORSEY, JR., B. S., M. D., Medicine
L. M. GAINES, A. B., M. D., Internal Medicine
GEO. C. MIZELL, M. D., Diseases of the Stomach and Intestines
L. B. CLARKE, M. D., Pediatrics
EDGAR PAULLIN, M. D., Opsonic Medicine
THEODORE TOEPEL, M. D., Mechano Therapy
E. G. BALLENGER, M. D., Diseases of the Genito-Urinary Organs

VOL. LXIV. ATLANTA, GA., JAN., 1918. NO. 10

### DOUBLE, RECURRENT AND BILATERAL TUBAL PREGNANCIES.

### An Analysis of 89 Cases Reported in the Literature and 3 Unpublished Personal Cases.

**Aime Paul Heineck, M.D., Chicago.**

Extra-uterine pregnancy is one of the most important maladies of the child-bearing period. It occurs in all races, appears to be less frequent in the colored, "4 negresses of 169 cases." The condition, though more frequently recognized than heretofore is, nevertheless, often overlooked, misdiagnosed and, therefore, mistreated. A more complete understanding of tubal gestation will lead to the saving of lives and to the prevention of invalidism.

Tubal gestation, by far the most common variety of ectopic pregnancy, is single, double, or multiple; unilateral or bilateral. It may be a woman's first and last conception; it may be preceded by a long period of infertility; it may end a woman's child-bearing career. Tubal pregnancy may coexist with a uterine pregnancy. It can occur in the absence of other pathological states of the pelvic or other organs. Its occurrence in one tube does not protect against its occurrence in the opposite tube, does not absolutely protect against its recurrence in the same tube.

To facilitate the task of future investigators, I have collected, studied and alalyzed all cases of double and bilateral tubal pregnancies reported with sufficient data in the English, French and German literature from 1908 to 1916, inclusive. Only original reports of cases in which the diagnosis was verified at operation were considered.

Double tubal pregnancies are almost invariably bilateral; exceptionally unilateral.

Double and bilateral pregnancies are either simultaneous or recurrent. If simultaneous, both conceptions begin at or about the same time; both gestations may develop, or one may be interrupted and the other continue. Usually, the two fetal cysts differ in size and destiny. The recurrent type is by far the most frequent (63 cases). Almost always, the recurrence is in the opposite tube.

Double and bilateral tubal pregnaicies can occur at any period of the child-bearing age. We do not know how often tubal pregnancy recurs; we do not know why it occurs. Authors are not agreed as to the frequency of recurrence.

One ectopic pregnancy is not necessarily followed by another ectopic pregnancy. Normal pregnancies may be sandwiched in between two extra-uterine gestations.

Months, or even years, may elapse between the incidence of pregnancy in one tube and the lodgment of an impregnated ovum in the opposite tube.

In our collected cases the interval between the two tubal gestations varied from three months to nine years.

Double, recurrent and bilateral tubal pregnancies occurred in women who have never borne living children. Tubal pregnancy has recurred in women who have borne one living child, two children, three, children, four children, five children and six children.

Double, recurrent and bilateral tubal pregnancies like other varieties of ectopic gestation not infrequently occur in women who though frequently exposed to pregnancy, have remained sterile.

The cause of tubal pregnancy, whether single, double or recurrent, is not definitely known. No causative factor present in every case has been demonstrated. Not uncommonly, co-existing pathological states are found. Are these pathological states coincidental or etiologic factors? With the data at hand, a positive answer is not possible.

Inflammatory and other degenerative changes of the tubal wall do not possess the important etiological role formerly attributed to them. Though all conditions that obstruct, delay or hinder the progress of the impregnated ovum to the uterus favor the occurrence of etopic gestation, still many cases occur in which the existing tubal gestation excepted, there is a total absence of pathological tubal or ovarian changes, congenital or acquired. Actual examination at time of operation has firmly established the fact that an inflammatory condition is not present in all cases.

It has been believed that the predominant cause of tubal pregnancy is salpingitis, post-abortum, post partum or gonorrheal in nature, with resulting destruction of the tubal ciliated opithelium. "I have been able to demonstrate the presence of cilia in nearly every pregnant tube which I have examined," Williams. In some cases, the presence of co-existing pelvic pathological states is recorded, cyst of parovarium, ovarian cyst, polycystic degeneration of left ovary.

All our collected and personal cases were primarily either interstitial, isthmic, or ampullary. All the others were bilateral. These 92 cases represent 185 tubal gestations. Not one of these pregnancies, either first or second, went to full term.

Sixteen gestations were subjected to operative relief previous to tubal abortion or tubal rupture. Thirty two tubal gestations terminated in abortions: seventy-five in rupture. In the remaining cases, the termination is either on trecorded or not befinitely stated. Termination dpends in great part upon the implanation site of the ovum, thus in the isthmic form, this portion of the tube not admitting of much distention, early rupture is the rule. In the ampullary form, the tubal wall offering less resistance in the ampullary region to the growth of the ovum, abortion is the rule. Tubal abortions are due to rupture through the capsular membrane; they are incomplete, or complete; incomplete being the more common. Complete tubal abortion implies complete expulsion of the ovum, membrane and contents, into the peritoneal cavity by way of the abdominal ostium of tube. In complete abortion, the hemorrhage is usually slight. In the incomplete type, there is a partial loosening of the ovum from the tubal wall and only parts of the ovum pass into the peritoneal cavity. In incomplete tubal abortion, the hemorrhages recur as evidenced by repeated appropriately designated by some authors as intra-tubal rupture.

Rupture, extratubal, occurs at or near the placental site, taking place either into the peritoneal cavity or between the folds of the broad ligaments. Primary rupture of the ovum, in by far the larger number of cases, occurs previous to or about the eighth week; in a few cases it occurs later.

It may involve any portion of the tube, isthmic, middle third, ampullary, and vary in size from a pin-point to a tearing asunder of the entire tube. Even a pin-point rupture may cause a fatal homorrhage. In the only case of this series in which hemorrhage apparently caused death, the rupture was a small orifice on the free portion of tube through which chorionic villi projected. The tubal tissues in contact with the ovum offer slight resistance to the fetal elements and being early invaded by the chorionic villi and fetal cells, they soon undergo degenerative changes. The tubal wall is weakened partly by the continuous and gradually increasing distention exerted by the growing ovum and much more so by the ercsive action of the fetal elements upon the maternal tissues. The tubal resistance being thus impaired, rupture is easily brought about either by direct perforation by the growing villi or by any sudden access of pressure, such as is determined by the sudden opening of a large vessel, by the clogging of vonous channels, or by slight external violence as vaginal examination, coitus, fall, etc.

Bilateral tubal gestation may terminate in tubal rupture in one tube and in tubal abortion in the other.

Tubal abortion and tubal rupture, be the latter intra or extra-tubal, are associated with moderate or profuse internal hemorrhage, either in the lumen of the Fallopian tube, between the folds of the broad ligament or into the peritoneal cavity.

When hemorrhage takes place into the free peritoneal cavity, a practically limitless space, the patient may bleed to death without a drop of blood appearing externally. Blood extravasated in the lumen of the tube, between the folds of the broad ligament or in the peritoneal cavity, either under goes absorption, coagulation, organization, cyst-formation, or suppuration.

**Fate of the Ovum.**

The ovum lodged in a tube being always poorly fixed, poorly nourished, most tubal pregnancies come to an end previous to the eighth week. When tubal gestation ends this early, be the termination due to ovular apoplexy, tubal abortion or tubal rupture, the ovum is absorbed. This is the fate of young embryos extruded into the peritoneal cavity, if they be not removed by the surgeon. When, after tubal abortion or tubal rupture, the placenta retains some tubal implantation and contracts new attachments to the pelvic wall, rectum or other viscus or viscera, the placental circulation thereby continuing, the pregnancy becomes tuba-abdominal or tubo-peritoneal type. Abortion is more difficult after the third month.

In many operations for early tubal gestation, the embryo is found in the tube or in the abdominal or peritoneal cavities. Ovular debris, placenta, decidual cells, fetal rests, chorionic villi, etc., are more frequently found at time of operation than fetuses.

The symptoms of tubal gestation, like those of uterine gestation can be classified into presumptive, probable and positive. The positive symptoms of pregnancy; fetal heart sounds, active and passive fetal movements, palpation of fetal parts, are usually not detected until after the fourth month of gestation. Now as 81 per cent. of tubal gestations terminate before, at or about their eighth week, it can be seen that the positive signs of tubal pregnancy, corresponding to the positive signs of uterine pregnancy, are rarely present and, therefore, rarely detected. In not one of our cases were any of the positive signs of pregnancy present.

Previous to tubal abortion and to tubal rupture, presumptive signs of pregnancy, such as amenorrhea, nausea and vomiting, bluish discoloration of vaginal walls, pigmentation and strias, urinary disturbances, were noted in many of the cases. Amenorrhea is so constant a symptom in tubal pregnancy that its absence is misleading.

Other presumptive symptoms such as nausea and vomiting, colostrum secretion, milk secretion, bluish discoloration of the vaginal wall, enlargement of breasts, etc., are less frequently recorded.

Among the probable signs, the most frequently noted in our series were changes in size, consistency and position of the uterus. "The existence of an enlarged uterus at any time during the child-bearing period should be regarded as presumptive evidence of pregnancy until such a possibility has been conclusively eliminated."—Williams.

The most characteristic symptoms that confront the clinician are those determined by tubal rupture or by tugal abortion. Both of these accidents are associated with pain and with internal hemorrhage, the extent of which determines the gravitiy of the case. Very often the patient first comes into the hands of the physician, some time after she has recovered from the primary shock due to tubal rupture or tubal abortion.

In tubal abortion there may be acute, severe, cramp-like pain, limited to the pelvic region or referred to other portions of the abdomen; there may be absence of pain. In many cases of tubal abortion about the only symptom we have is abdominal pain and uterine colic preceding and accompanying the expulsion of the decidual cast. In tubal rupture, the pain is intense, agonizing, may cause the patient's collapse. It is most marked in the lower abdomen and may be referred to the right side, to the left side, to right kidney region, to the rectum, epigastrium, umbilicus.

Coincident with the lodgment and development of the ovum, the uterus, during the first three months of tubal gestation, undergoes hypertrophy and its endometrium becomes converted into a decidua similar to that observed in uterine pregnancy. Soon after the death of the fetus, the decidual is thrown off, being expelled in shreds, or as a triangular cast of the uterine cavity, with dimensions corresponding to that of the hypertrophied uterus.

Though tubal pregnancy and especially bilateral tubal pregnancy, are frequently operative discoveries, the diagnosis being rarely made previous to tubal abortion or tubal rupture, the following symptoms, taken in conjunction with a suggestive history and suggestive pelvic findings, should make one think of the possible existence of tubal gestation.

a. Presence of the presumptive symptoms and signs of pregnancy: Morning sickness, milk and colostrum secretion, pelvic pains referable to bladder and rectum.

b. Cessation of the menses.

c. Bluish discoloration of the vaginal wall.

d. Softening of the cervix.

e. Changes in size, consistency and position of uterus.

The existence of ectopic pregnancy is highly probable, when, in association with the above, palpation reveals an indefinitely outlined tender, boggy mass to one or both sides of uterus, in a patient who has or has had symptos of acute anemia and attacks of acute abdominal pain, especially if the abdominal tumor has increased in size with each attack of abdominal pain.

If, during an intermenstrual period with or without a suppression of the menses, a woman has a nattack of sever abdominal pain followd by vomiting, collapse, slight uterine hemorrhage, think of tubal abortion. If after a few days or a few weeks, the same clinical picture recurs, suspects the existence of a bilateral tubal prègnancy.

The severe pain of tubal rupture is accompanied or followed by symptoms of obdominal hemorrhage and acute anemia, pallor, dizziness, nausea, collapse, weak thready pulse. In almost all cases associated with the above, vaginal hemorrhage varying in amount, slight, or profuse, and in duration, from 3 weeks to 6 weeks, is said to have been present. These attacks of pain, vaginal hemorrhage and anemia may be repeated.

The treatment of ectopic gestation previous to, at time, of, or after tubal rupture or abortion is operative. As stated in some of our previous publications on this subject, we disregard completely the life of the ectopic fetus and concentrate our efforts to saving the maternal health and maternal life. The ectopic fetus, in all its various forms and at all periods of its existence, is a menace to the maternal organism. Operation removes in a few minutes what it will require unaided nature, even in the most favorable cases, a long time to accomplish and thereby early secures the safety of the patient.

The operation for the relief of ectopic pregnancy, for the control of its complications and the cure of its sequelae, may be an emergency operation, may be one giving us time for ample preparation of the patient. In a general way it can be said that an ectopic gesttion is a malignant growth and the longer it is unmolested, the greater are the dangers to the mother.

In cases of tubal rupture and also in cases of tubal abortion associated with symptoms of abdominal hemorrhage, operative relief must be immediately instituted. A patient can bleed to death into the peritoneal cavity without a drop of blood appearing externally. Peritoneal flooding calls for immediate intervention. Operation is equally indicated previous to tubal abortion or tubal rupture but under these conditions if the patient be vigilantly watched, a delay of two or three days is not very significant.

In all operations for ectopic pregnancy, we discard the vaginal route. We prefer the abdominal route. We are justified in making our diagnoses and basing our management of cases upon presumptive evidence. A large mortality results from delayed diagnoses.

The most immediate danger of tubal abortion or tubal rupture is hemorrhage. Laparotomy permits an immediate and complete arrest of hemorrhage. Laparotomy not only secures absolute hemostosis but enables one to eliminate the danger of post-operative or secondary hemorrhage. It permits a more complete removal of the ovular debris and extravasated blood. It is not necessary to remove all blood from the peritoneal cavity. Let there be no needless treumatizing. Furthermore, laparotomy allows inspections of the pelvic organs and enables one to decide at once whether or not the opposite tube should be removed.

Unilateral tubal pregnancy calls for removal of the pregnant tube. The operator must not be haunted by the thought of recurrence. Recurrence in the opposite tube is exceptional.

We are not justified in sterilizing a woman just because she has sad a tubal gestation.

Remove the unaffected tube:

a. If there be existing in the patient some constitutional state contra-indicating pregnancy such as epelepsey, alcoholism, worst types of neurasthenia, syphilis, mental disease, imbecility, advanced tuberculosis, advanced cardiac, renal, or hepatic disease, bad types of primary anemia.

b. If there be existing in the patient some pelvic deformity preventing delivery through the maternal passages of a viable fetus.

c. If it be imbedded in adhesions, if it be malformed or the seat of a congenital anomaly or of inflammatory, neoplastic or other degenerative changes; hydrosalpinx, pyosalpinx, etc.

In unilateral tubal pregnancy and in bilateral tubal pregnancy there should be no needless removal of tissues or organs. Therefore, if the ovaries are normal or only slightly altered, their preservation will be of great benefit to the patient. In addition to removing pregnant tube, fetus and ovular debris, correct co-existing pathological states if the patient's condition permits.

The mortality of bilateral tubal pregnancy, skillfully operated on, is very low. It should be nil. In our collected cases, there were only three deaths; two from peritonitis and ileus and one from peritoneal hemorrhage.

